# Evaluation of Effects of Intraperitoneal CO_2_ Pressure in Laparoscopic Operations on Kidney, Pancreas, Liver and Spleen in Dogs

**DOI:** 10.5812/ircmj.7805

**Published:** 2013-09-05

**Authors:** Mohamad Hejazi, Mir sepehr Pedram, Hosein Ashegh, Nazanin Jafari, Fereshteh Ghazisaeedi, Mahboobeh Abdi

**Affiliations:** 1 Department of Animal Science, Agriculture Faculty, Islamic Azad University, Khorramabad Branch, Khorramabad, IR Iran; 2 Department of Clinical Science, Faculty of Veterinary Medicine, University of Tehran, Tehran, IR Iran; 3 Laparoscopy Training Center, Tehran University of Medical Science Tehran, IR Iran

**Keywords:** Laparoscopy, Pneumoperitoneum, Kidney, Liver, Pancreas, Spleen

## Abstract

**Background:**

During laparoscopy, insufflation of an inert gas in the peritoneal cavity creates a working space to facilitate surgery. The space should be large enough to facilitate surgery without increasing intra-abdominal pressure (IAP) over a threshold limit (usually 15 mm Hg).

**Objectives:**

This experimental study was performed to evaluate the effects of increasing in intra-abdominal pressure on internal organs.

**Materials and Methods:**

Twenty female mixed breed dogs (20 ± 3 kg, 18 ± 1.2 months) were selected. They were randomly divided to two groups (n = 10). The intra-abdominal pressure was maintained 12 mm Hg and 20 mm Hg during the operation in control group and in test group respectively.

**Results:**

Histopathologic evaluations revealed more pathological changes at the kidney of all the dogs in test group in comparison to control group.

**Conclusions:**

Our findings revealed that organs that their blood supplies are related to one single or two arteries and their blood drainage are related to one or two veins are more sensitive to increased intra-abdominal pressure.

## 1. Background

Creation of working space by insufflating an inert gas during laparoscopy is essential in most procedures to facilitate surgery (1). The space should be large enough to facilitate surgery but it is mostly accepted not to reach over a threshold limit (usually 15 mm Hg) (1). Carbon dioxide has been commonly used to induce pneumoperitonium in laparoscopic operations. The valuable advantages make this gas widely preferable in laparoscopy surgeries, such as its high solubility and rapidly absorption in the blood, therefore minimizing the risk of gas embolism, (1, 2) and lose of the risk of combustion, (2) the possibility of permitting a safe electro cautery, (1) make CO_2_ a suitable and reliable gas for induction the space in abdominal cavity in laparoscopic operations. Although insufflate the intra-peritoneal space with CO_2_ have some potential disadvantages such as abdominal injuries and cardiovascular and respiratory disorders, but with exception of some critically ill patients, this procedures is widely accepted (1).

Most of laparoscopic complications occur during the entrance of trocars into the abdominal cavity. Indeed at least 50% of all complications at laparoscopic operations are related access technique and take place before the operation commences (3, 4). For decreasing the risk of entry-related injuries several methods have been explored over 50 years and no technique has been recognized as the safest access technique (5-9). One of the methods that described to surmount this complication is inducing peritoneal hyper-distention (10, 11). In this technique before the insertion of the first trocar, the intra-abdominal pressure reaches as much as 25 – 30 mm Hg by using a Veress needle (10). This technique creates greater space and therefore decreases the risk of inducing damages to the viscera by trocars. The intra-abdominal pressure reduces to 15 mm Hg immediately after entrance of trocars (4, 6, 10-12). It is important to mention that the greater intra-abdominal space will reduce the risk of complications during the entrance of trocars and during the operation (10, 11). Nowadays the important question is that “what happens if the intra-abdominal pressure remains in high levels?” Although there are some studies that revealed no significant cardiopulmonary effects in high intra-abdominal pressure, (12) the high pressure technique has not gained popularity. One possible reason is anesthetic concerns that may be influenced by high intra-peritoneal pressure (9, 13-17).

## 2. Objectives

The purpose of this study was to evaluate the pathological effects of intra-abdominal high-pressure (20 mmHg) on the viscera including Kidney, Pancreas, Spleen and Liver during laparoscopic operations.

## 3. Materials and Methods

Twenty female mixed breed dogs which were chose for an experimental procedure were prepared. Average of weight was 20 ± 3 kilograms and average of age was recorded 18 ± 1.2 months. Clinical and biochemical exams before operation were performed and did not show any sign of diseases or disturbances. All experiments were performed according to European Animal Care Committee guidelines. Dogs were used in laparoscopy training center of Tehran University of Medical Science as a model and undergoing a Cholecystectomy procedure with no technical complications and suffered operation with no mortality. They were randomly divided to two groups (n = 10). Acepromazine was administrated in all dogs (KELA Laboratoria NV. Hoostraten / Belgum) (0.05 mg/Kg) by intramuscular route as premedication. Induction of anesthesia was accomplished by intravenous injection of ketamine (10 mg/kg) (alfasan Woerden-Holland) and Diazepam (Dr. Amidi Ins. Iran) (0.2 mg/kg). For maintenance of anesthesia, slow infusion of ketamine (0.1 mg/kg/h) was used. All animals were treated pre-operativly with ketoprofen (2 mg/kg, IV). Laparoscopic procedure and induction of insufflation was performed with Richard WOLF devices and instruments.

In control group the intra-abdominal pressure was maintained 12 mm Hg and in test group 20 mm Hg during the operation (Insufflator: Richard WOLF 2233). This condition was maintained at least for 4 hours. The dogs were euthanized with IV injection of pentobarbital sodium and samples were taken from liver, pancreas, kidney and spleen for histopathologic evaluations. After standard fixation of the samples and preparation of the histological blocks, H&E staining was performed and several factors were evaluated.

Data were compared between control and test groups. Effects of intra-abdominal pressure on the mentioned tissues were evaluated by histopathologic indexes and the data were analyzed using an ANOVA for repeated measures.

## 4. Results

There were no differences between dogs in body weight, age and the procedure of general anesthesia. In heart rate evaluation at equal intervals, no significant differences were observed over time between the two groups. Also in respiratory rate, there were no significant differences (P>0.05). Results of end tidal CO_2_ measurements did not show severe hypoventilation during anesthesia in both groups. Comparison of this data between groups did not show any significant difference (P > 0.05). The mean of hemoglobin saturation measurements in the two groups did not show any significant difference (P > 0.05).

**Figure 1.**
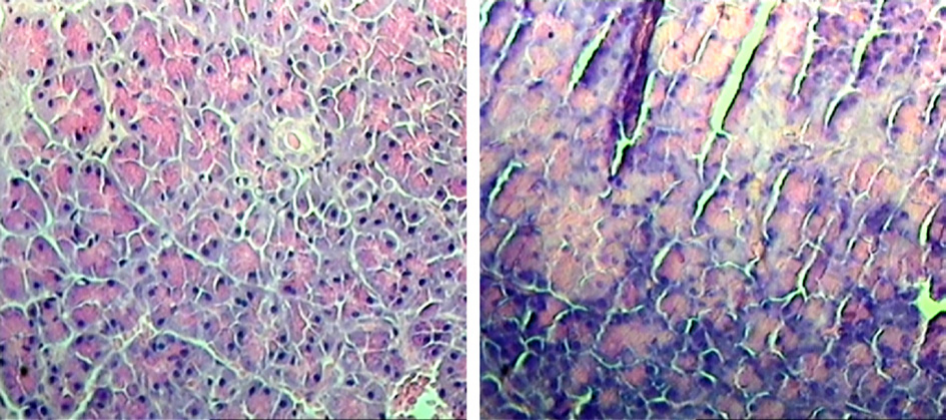
Pancreas Acinar Necrosis (a) normal acinar histological section of pancreas (control group), (b) acinar necrosis histopathological section of pancreas (test group; intraabdominal pressure: 20 mm Hg).

**Figure 2.**
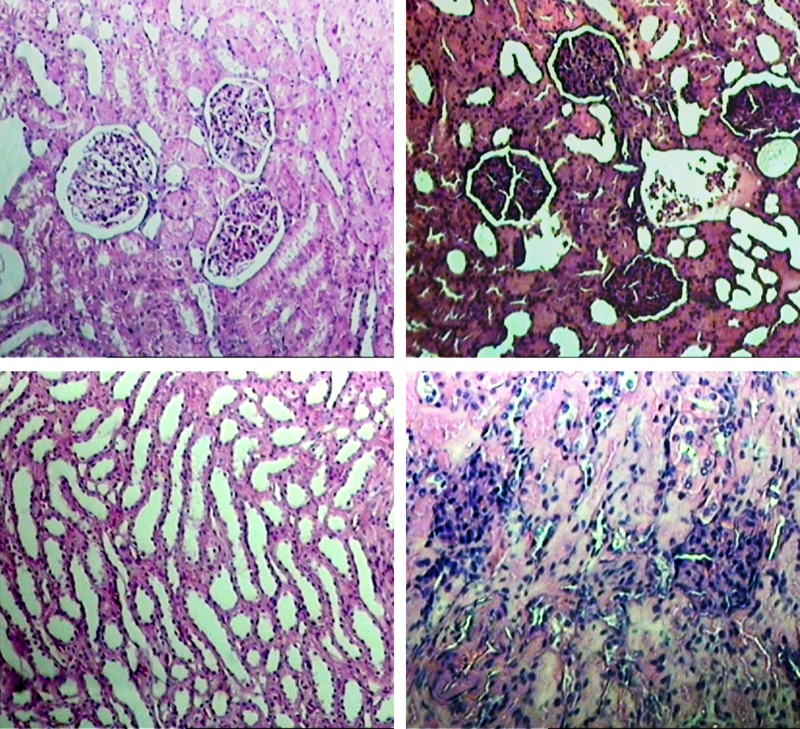
Kidney Histopathological Changes (a) Normal renal cortex histological section (control group), (b) necrosis of renal cortex (test group; intraabdominal pressure: 20 mm Hg), (c) normal medullary tubules (test group; intraabdominal pressure: 20 mm Hg), (d) more sever necrosis of renal cortex (test group; intraabdominal pressure: 20 mm Hg).

Histopathologic evaluations revealed pathological changes at the kidney of all the dogs in test group (intraabdominal pressure: 20 mm Hg) (Figure 1) in comparison to control group (intra-abdominal pressure: 12 mm Hg) (Figure 2). These pathological changes included: coagulation necrosis, glomerulonephritis and fatty changes. Fatty changes were stronger at distal tubules in comparison with proximal tubules. 

The pathologic changes at the liver were similar in both groups and were mild. These changes included mild coagulation necrosis in the parenchyma, hyperplasia at the smooth muscles and congestion. The differences between two groups were not significant. The pathologic change that was significant in test group at the pancreases was acinar necrosis. This change was observed only in samples of test group. Any pathologic change at the spleen was not detected in two groups.

## 5. Discussion

Nowadays laparoscopy procedures are performed widely all over the world for several purposes. It is essential to evaluate different aspects of this procedure and investigate about the possible complications in experimental studies. One of the most important aspects of laparoscopy procedures is intra-abdominal pressure during the operation.

Although Arterial PaO_2_, oxygen saturation, and end tidal Co_2_ remained unchanged in low and high levels of IAP, Yavuz et al. showed that in 24 mmHg tissue blood flow was significantly decreased in some organs such as spleen, pancreas, esophagus, and gastric mucosal in pigs (18) The present study investigates if this decrease of blood flow may cause degenerative changes in these organs.

Although Hung et al. in 1995 revealed that different limits of intra-abdominal pressure routinely used during laparoscopic surgery did not affect metabolic function, acid-base balance, or hemodynamics in the experimental model but absorption of Co_2_ across the peritoneum during the laparoscopic operations may affect vital organs by introduction of acidemia, hypercapnea, and depressed hemodynamics (19).

The present study showed that increase of intraabdominal pressure during the operation may cause pathological effects on internal organs. Although hemodynamic changes in kidney are mostly transient and reversible after a period of two hours in normal limits of intra-abdominal pressures, these degenerative changes may affects the kidneys permanently at high pressures (20). Three factors that may affect hemodynamic of kidney as an important organ during high levels of intra-abdominal pressure in laparoscopic surgeries, are local compressing effect, decreased cardiac output, and decreased venous return. Mild effects on renal parenchymal perfusion are probably related to local compressing effect during pneumoperitonium (21). Besides these effects, concentration of endothelin after renal vein compression that occurres during pneumoperitonium may elevate at high levels of intra-abdominal pressure and may contribute to oliguria (22). It is important to mention that intra-abdominal pressure has more influence on organs than type of the gas used for insufflations (23).

Present study showed that increased intra-abdominal pressure has pathological effects mostly on kidney and then pancreas. Also this study revealed that increased intra-abdominal pressure does not have any significant effects on liver or spleen. These finding showed that organs that their blood supplies are related to one single or two arteries and their blood drainage are related to one or two veins are more sensitive to increased intra-abdominal pressure.
